# Assessing the impact of ecological, climatic, and socioeconomic factors on age-specific malaria incidence in India: a mixed-model approach using the Global Burden of Disease Study (2010–2019)

**DOI:** 10.1186/s12936-024-05151-2

**Published:** 2024-11-08

**Authors:** Bijaya K. Padhi, Abhay M. Gaidhane, Prakasini Satapathy, Ganesh Bushi, Suhas Ballal, Pooja Bansal, Balvir S. Tomar, Ayash Ashraf, M. Ravi Kumar, Pramod Rawat, Mohammed Garout, Fatimah S. Aljebaly, Amal A. Sabour, Maha A. Alshiekheid, Nawal A. Al Kaabi, Hayam A. Alrasheed, Maha F. Al-Subaie, Ali A. Rabaan, Ahmed Saif, Sarvesh Rustagi, Quazi Syed Zahiruddin, Muhammed Shabil

**Affiliations:** 1 Department of Community Medicine and School of Public Health, Postgraduate Institute of Medical Education and Research, Chandigarh, 160012 India; 2 Jawaharlal Nehru Medical College, and Global Health Academy, School of Epidemiology and Public Health, Datta Meghe Institute of Higher Education, Wardha, India; 3 Center for Global Health Research, Saveetha Medical College and Hospital, Saveetha Institute of Medical and Technical Sciences, Saveetha University, Chennai, India; 4 Medical Laboratories Techniques Department, AL-Mustaqbal University, 51001 Hillah, Babil, Iraq; 5 School of Pharmaceutical Sciences, Lovely Professional University, Phagwara, India 144411; 6 Department of Chemistry and Biochemistry, School of Sciences, JAIN (Deemed to Be University), Bangalore, Karnataka India; 7 Department of Allied Healthcare and Sciences, Vivekananda Global University, Jaipur, Rajasthan, 303012 India; 8 Institute of Pediatric Gastroenterology and Hepatology, NIMS University, Jaipur, India; 9 Chandigarh Pharmacy College, Chandigarh Group of College, Jhanjeri, Mohali, 140307 Punjab India; 10 Department of Chemistry, Raghu Engineering College, Visakhapatnam, Andhra Pradesh 531162 India; 11 Department of Biotechnology, Graphic Era (Deemed to Be University), Clement Town, Dehradun, 248002 India; 12 Department of Allied Sciences, Graphic Era Hill University, Clement Town, Dehradun, 248002 India; 13 Department of Community Medicine and Health Care for Pilgrims, Faculty of Medicine, Umm Al-Qura University, 21955 Makkah, Saudi Arabia; 14 Department of Basic Medical Sciences, Unaizah College of Medicine and Medical Sciences, Qassim University, 51452 Qassim, Saudi Arabia; 15 Department of Botany and Microbiology, College of Science, King Saud University, 11451 Riyadh, Saudi Arabia; 16 College of Medicine and Health Science, Khalifa University, 127788 Abu Dhabi, United Arab Emirates; 17 Sheikh Khalifa Medical City, Abu Dhabi Health Services Company (SEHA), 51900 Abu Dhabi, United Arab Emirates; 18 Department of Pharmacy Practice, College of Pharmacy, Princess Nourah Bint Abdulrahman University, 11671 Riyadh, Saudi Arabia; 19 Research Center, Dr. Sulaiman Alhabib Medical Group, 13328 Riyadh, Saudi Arabia; 20 College of Medicine, Alfaisal University, 11533 Riyadh, Saudi Arabia; 21 Molecular Diagnostic Laboratory, Johns Hopkins Aramco Healthcare, 31311 Dhahran, Saudi Arabia; 22 Department of Public Health and Nutrition, The University of Haripur, Haripur, 22610 Pakistan; 23 Department of Clinical Laboratory Sciences, College of Applied Medical Sciences, King Khalid University, Abha, Saudi Arabia; 24 School of Applied and Life Sciences, Uttaranchal University, Dehradun, Uttarakhand India; 25 South Asia Infant Feeding Research Network (SAIFRN), Division of Evidence Synthesis, Global Consortium of Public Health and Research, Datta Meghe Institute of Higher Education, Wardha, India; 26 University Center for Research and Development, Chandigarh University, Mohali, Punjab India

**Keywords:** Malaria incidence, Climatic variables, Socio-economic factors, Generalized linear mixed models, Human development index, Public health strategies, SDG

## Abstract

**Background:**

Malaria continues to be a critical public health concern in India, predominantly driven by complex interplays of ecological, climatic, and socioeconomic factors.

**Methods:**

This study aimed to assess the association between climatic variables (temperature and precipitation) and malaria incidence across India from 2010 to 2019, utilizing data from the Global Historical Weather and Climate Data for climate metrics and the Global Burden of Disease Study for malaria incidence rates. Generalized Linear Mixed Models (GLMMs) with a Poisson distribution were employed to analyze the data, adjusting for socio-economic status, as indexed by the Human Development Index (HDI).

**Results:**

The results indicated a declining trend in both the number of malaria cases and age-specific incidence rates (ASIR) over the study period. In 2010, India reported approximately 20.7 million cases with an ASIR of 1688.86 per 100,000 population, which significantly reduced to 9.8 million cases and an ASIR of 700.80 by 2019. High malaria incidence was consistently observed in the states of Jharkhand and Odisha, whereas Sikkim reported the lowest numbers. Statistical analysis identified significant associations between malaria incidence and both temperature deviations and precipitation levels, with variations also linked to HDI, suggesting better detection and reporting capabilities in more developed areas.

**Conclusion:**

The study underscores the critical interactions between climatic variables and socio-economic factors in shaping the trends of malaria incidence across India. These findings highlight the necessity for adaptive, localized public health strategies that integrate environmental monitoring with socio-economic data to efficiently predict and manage malaria outbreaks.

## Background

Malaria stands out as one of the most serious and complex global public health problems. Despite the rigorous and continuous efforts of various health organizations, it remains a significant challenge, impacting people’s health and livelihoods worldwide. In addition, it remains among the top causes of disease and mortality in sub-Saharan Africa even though it is preventable and treatable [1]. Across 85 malaria-endemic nations and regions globally, there were almost 249 million cases of malaria in 2022—a 5 million increase over the previous year. Key contributors to this increase included Pakistan, Ethiopia, Nigeria, Uganda, and Papua New Guinea. Even though African nations constituted roughly 94% of the total global malaria cases in 2022, totalling around 233 million cases, it's crucial to recognize the malaria burden in other regions. The World Health Organization's Southeast Asia region (SEAR) contributed approximately 2% of the global malaria cases and experiencing an estimated 83% decline in malaria incidence from 2000 to 2022 [1]. Within the SEAR, India contributed to 66% of the malaria cases, while collectively, India and Indonesia accounted for roughly 94% of all malaria deaths in the region.

In India, a pivotal moment in the understanding of malaria occurred when Roland Ross, a British Army Officer serving in the Indian Medical Service, declared on August 27, 1897, that he had proven mosquitoes could transmit malaria. His discovery demonstrated that mosquitoes became carriers of the disease after feeding on a person infected with the malaria parasite and subsequently biting an uninfected individual. This revelation highlighted the crucial role of mosquitoes in the spread of malaria and sparked interest among public health experts in the potential for malaria elimination strategies. In 1935, India faced a staggering burden of malaria, with an estimated 100 million cases and one million deaths annually [2]. However, a significant turnaround occurred with the initiation of the National Malaria Eradication Programme in 1958. By the mid-1960s, the death toll plummeted to zero, with only 0.1 million reported cases, marking a remarkable achievement in virtually eliminating the disease from the country [3, 4]. This success bred complacency, leading to a belief that malaria had been conquered. Yet, challenges emerged in the 1970s due to factors like dichlorodiphenyltrichloroethane (DDT) resistance, resulting in a resurgence of the disease. Reported cases spiked to 6.5 million in 1975 [4, 5]. Despite subsequent reductions to 2.38 million during 1990–2000 and further declines to 0.79 million annually from 2011 to 2022, malaria remains a persistent health concern in India [6].

Malaria is the result of a parasite transmitted to humans by mosquitoes of the *Anopheles* genus. These mosquitoes, seeking blood for their egg production, transmit the *Plasmodium* parasite while feeding [7]. The development and maturation of this parasite are largely influenced by climatic conditions such as temperature, rainfall, and relative humidity [8, 9]. Therefore, any alterations in these climate factors would undoubtedly impact mosquito ecology [10–12]. Of all vector-borne diseases influenced by environmental and climatic factors, malaria imposes the largest burden worldwide. This gives rise to legitimate concerns about how attempts to combat the disease may be impacted by climate change [13–15]. The impact of climate change on malaria transmission is subject to diverse viewpoints. While some experts anticipate a significant expansion of malaria, others argue that the direct influence on transmission may be minimal, particularly considering changes in other contributing factors [16–20]. Furthermore, some studies indicate a potential reduction in malaria burden in response to climate change [21]. Despite these differing perspectives, there is agreement that the interaction between climate change and malaria transmission is intricate, and there is limited empirical evidence to provide reliable predictions [15, 19, 22]. This underscores why the impact of climatic and environmental variables on malaria incidence has been a central focus of research [23].

Multiple studies conducted worldwide indicate that climate plays a crucial role in determining the distribution of malaria vectors and pathogens [24–28]. Result obtained from certain studies suggest that rather than an immediate impact of climatic factors on malaria incidence, there may be a delayed effect [29, 30]. For instance, it has been noted that malaria incidence in Sri Lanka lags by zero to three months behind rainfall [31]. Temperature, rainfall, and humidity intricately influence malaria transmission [15, 17, 32]. These factors define the disease’s geographic boundaries as well as its seasonality and intensity within those boundaries [33, 34]. Malaria transmission peaks in regions where temperature, rainfall, and humidity are close to optimal levels, as commonly observed across extensive areas of sub-Saharan Africa as well as in many parts of India [14, 25, 35, 36]. Changes in these factors due to climate change are anticipated to impact malaria transmission and control efforts. The effects will be non-linear and context-dependent, varying with factors like malaria control measures, socioeconomic development, and environmental management [15, 18]. Extreme weather events caused by climate change, such as flooding and droughts, further complicate malaria dynamics [15].

India is working towards eliminating malaria throughout the country by 2030, as outlined in the Global Technical Strategy for Malaria 2016–2030 (GTS) [37]. The objectives include eliminating malaria from all low and moderate transmission states/union territories by 2022, reducing malaria incidence to less than 1 case per 1000 population annually in all states and UTs by 2024, interrupting indigenous transmission nationwide by 2027, and preventing the re-establishment of local transmission to maintain a malaria-free status by 2030 and beyond [38]. The malaria reduction rate in India remains unsatisfactory despite ongoing programmes, exacerbated by limited state-specific research and challenges posed by global and national climate change. Existing literature on the impact of temperature and precipitation on malaria is inconclusive, with a lack of nation and state-level studies. This study aims to assess the association between national and state-level temperature, precipitation, and malaria incidence from 2010 to 2019 in India. By analyzing a decade of data, the study provides a comprehensive understanding of temperature and precipitation patterns and their relationship with malaria incidence. These findings are intended to complement previous research, enhance understanding of climate-malaria dynamics, and support malaria control efforts in the context of climate change, ultimately contributing to India's national and state-specific goal of malaria elimination.

## Methods

### Study design and data sources

This national observational study covered all states and union territories, which recorded malaria incidence along with monthly temperature and precipitation data from 2010 to 2019. Meteorological data were obtained from the Global Historical Weather and Climate Data (GHWCD), a comprehensive resource that provides weather and climate data (GHWCD, 2019). This collects records of temperature, precipitation, and atmospheric pressure. The collection of temperature, precipitation, and atmospheric pressure records in Global Historical Weather and Climate Data involves standardized procedures to ensure accuracy and consistency. Temperature is typically measured using thermometers placed inside Stevenson screens to shield them from direct sunlight and precipitation while allowing air flow for accurate ambient readings. Precipitation is captured using various types of rain gauges, including tipping bucket and weighing gauges, which automatically record the amount of rainfall. Atmospheric pressure is gauged with mercury and aneroid barometers, which measure pressure changes that are indicative of weather conditions. These instruments are routinely calibrated and maintained under guidelines set by the World Meteorological Organization. Data collected are then transmitted to databases where they undergo quality control, including homogenization to adjust for any discrepancies caused by changes in measurement methods or environmental factors, ensuring that the historical data are reliable and useful for research and forecasting purposes.

The Global Burden of Disease Study 2019, provides a comprehensive analysis of the various burden of disease on health over time, classified by gender, age, and geographical region [39]. The malaria incidence data utilized in this study is drawn from this comprehensive analysis.

### National annual average temperature and precipitation

Monthly temperature and total precipitation data for a designated area from the Global Historical Weather and Climate Data website, covering all months within a year. The data from all relevant meteorological observation regions were then combined and analysed on an annual basis. Finally, the annual temperature and total precipitation data for national meteorological regions were compiled, covering the period from 2010 to 2019.

### Malaria incidence data

Annual cases and incidence rates of malaria from 2010 to 2019, categorized by sex and age for India, were obtained from the Global Health Data Exchange, established and maintained by the Institute for Health Metrics and Evaluation (IHME). To account for differing age structures across populations and over time, age-standardized incidence rates (ASIRs) of malaria were also gathered. These rates were calculated by applying age-specific rates to a GBD world standard population. Sub-analyses were conducted by age categories (< 5, 5–14, 15–49, 50–69, and ≥ 70 years). The methodological techniques used to calculate the prevalence of malaria infection have been detailed in previous studies [39]. In summary, GBD standardized and consolidated all available incidence data into a unified database, which was then used to generate cause-specific estimates by age, sex, year, and geography [39].

### Statistical analysis

The annual average temperature and precipitation data for each state were computed using monthly temperature and total precipitation values from all monitoring stations. Annual temperature and precipitation maps were then generated using ArcGIS version 10.3.

The relationship between environmental conditions, socio-economic status, and malaria incidence was modelled using a Generalized Linear Mixed Model (GLMM) with a Poisson distribution, suitable for count data such as incidence rates. The model incorporated both fixed and random effects to account for observed and unobserved variability in the primary outcome variable, age-specific incidence rate (ASIR) of malaria per 100,000 population. Key variables included in the model were the States of India, year of data collection (2010, 2015 and 2019), average temperature (°C) during the year, total precipitation (mm) for the year, and the human development index (HDI) value of the states in these years. The dataset was subjected to pre-processing to align with the analytical needs, including normalization and transformation of certain variables.

### Data transformation

Transformations applied to the data were as follows:

Temperature deviation: This variable was calculated as the absolute value of the difference between the observed temperature and a baseline of 28 °C, which represents a typical mean temperature conducive to mosquito viability and malaria parasite development.

ASIR rounding (ASIR integer): The ASIR values were rounded to the nearest integer using the ceiling function to facilitate modelling with a Poisson distribution, which requires count data.

Normalized precipitation: Precipitation data were standardized by subtracting the mean and dividing by the standard deviation across all observations to reduce variability and improve model stability.

### Model specification

$${\text{log}}\left( {\lambda_{ij} } \right)\, = \,\beta_{0} \, + \,\beta_{{1}} \, \times \,Temperature\_Deviationij\, + \,\beta_{{2}} \, \times \,Normalized\_Precipitation_{ij} \, + \,\beta_{{3}} \, \times \,HDI_{ij} \, + \,u_{i} \, + \,v_{j}$$

where: λ_ij_ represents the expected ASIR for the ith state in the jth year; β_0_, β_1_, β_2_ and β_3_ are the fixed effect coefficients corresponding to the intercept, temperature deviation, normalized precipitation and HDI, respectively; u_i_ is the random effect for the ith state, capturing unobserved heterogeneity among different states; v_j_ is the random effect for the jth year, accounting for variations across years that affect all states similarly; log(*λ*_*ij*_) denotes the natural logarithm, linking the linear predictors to the expected age specific malaria incidence rates.

Intercept β_0_ represents the log ASIR when all predictors are at their mean values (baseline conditions). The coefficient of temperature deviation measures the impact of deviation from optimal malaria transmission temperature (28 °C) on ASIR. The coefficient of Normalized Precipitation assesses how variations in precipitation relative to the norm affect malaria incidence. The coefficient of the Human Development Index (HDI) evaluates the influence of socio-economic development on ASIR of malaria per 100,000 population. State-specific variability (u_i_) allows for non-constant variance across states due to unmeasured factors like local malaria control initiatives or microclimatic conditions. Year-specific variability (v_j_) captures annual fluctuations that might be due to factors like epidemic outbreaks or significant weather events.

### Model assumptions and performance evaluation

Model assumptions were verified by examining the residual plots and the significance of fixed effects was tested using Wald z-tests, provided by default in GLMM outputs. Variance Inflation Factor (VIF) and pair-wise correlation coefficients were calculated to assess the existence of a multicollinearity problem. The Akaike Information Criterion (AIC) and Bayesian Information Criterion (BIC) are statistical metrics used to evaluate model fit and complexity. Both criteria aim to resolve the trade-off between goodness of fit and model simplicity, penalizing the addition of unnecessary parameters. AIC focuses on information loss minimization, whereas BIC incorporates a stronger penalty for models with more parameters, especially as sample size increases. The marginal R-squared (R^2^_m_) value was assessed to quantify the variance explained solely by the fixed effects, while the conditional R-squared (R^2^_c_) measure was calculated to quantify the variance explained by both fixed and random effects combined.

## Results

### Malaria burden in India: national and state-specific trends (2010, 2015, 2019)

**Fig. 1:**
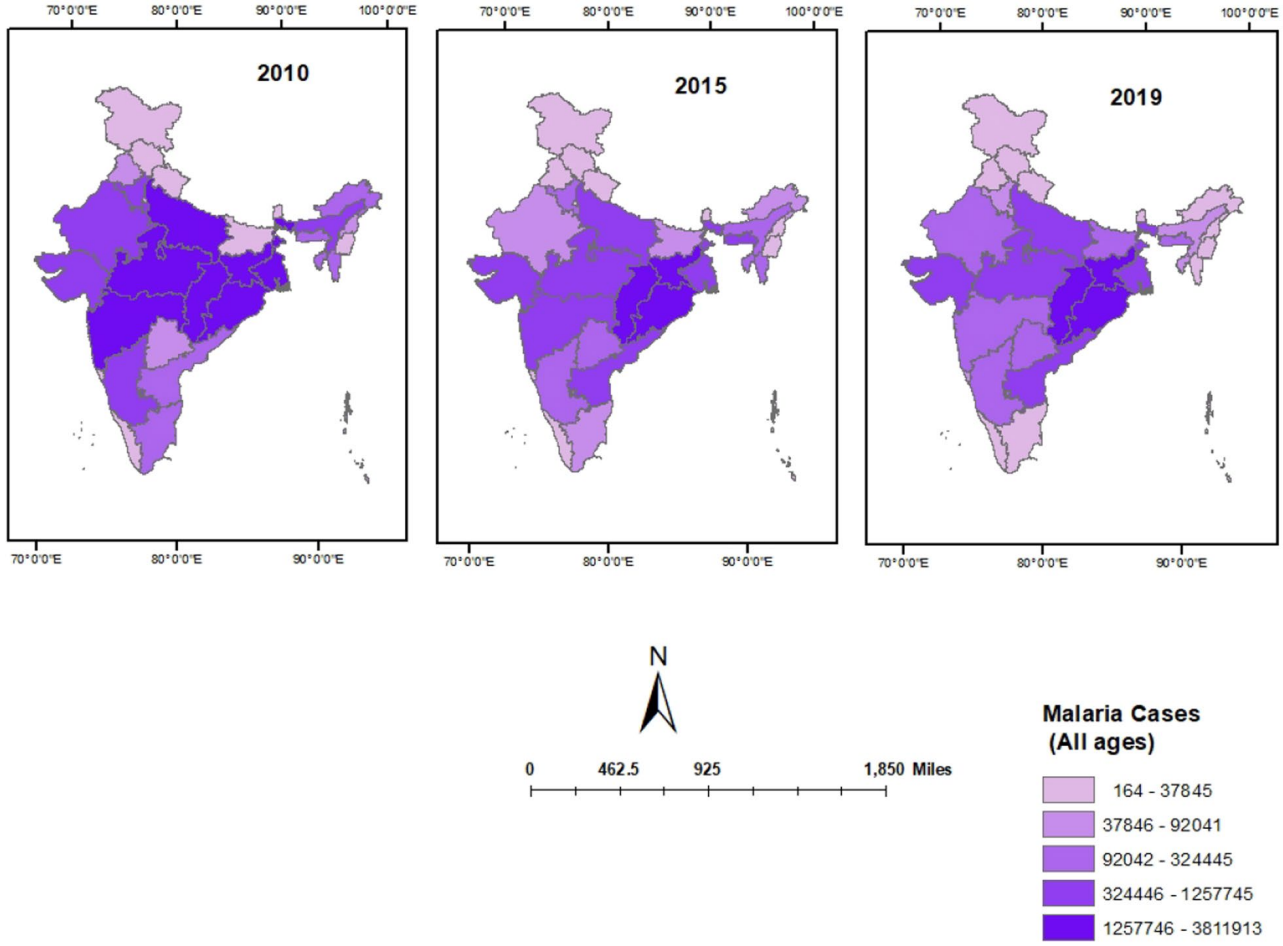
State-wise map of malaria cases in different states of India in 2010, 2015, and 2019

In 2010, nationally there were 20.7 million malaria cases with an ASIR of 1688.86 per 100,000 population. This decreases to 12.4 million malaria case and ASIR of 930 per 100,000 population in 2015. Similar pattern follows for 2019, malaria cases decrease to 9.8 million and relative ASIR decreases to 700.80 per 100,000 population. Jharkhand had the highest number of cases in 2010 38.11 million followed by Odisha 32.9 million and lowest in Sikkim with 3 × 10^–3^ million cases followed by Himanchal Pradesh with 1.7 × 10^–2^. Odisha had highest number of cases in 2015 and 2019 (29.7 million and 19.5 million respectively) while Sikkim is the lowest malaria cases in 2015 1.6 × 10^–3^ and 2019 4.4 × 10^–4^ (Fig. 1).

**Fig. 2:**
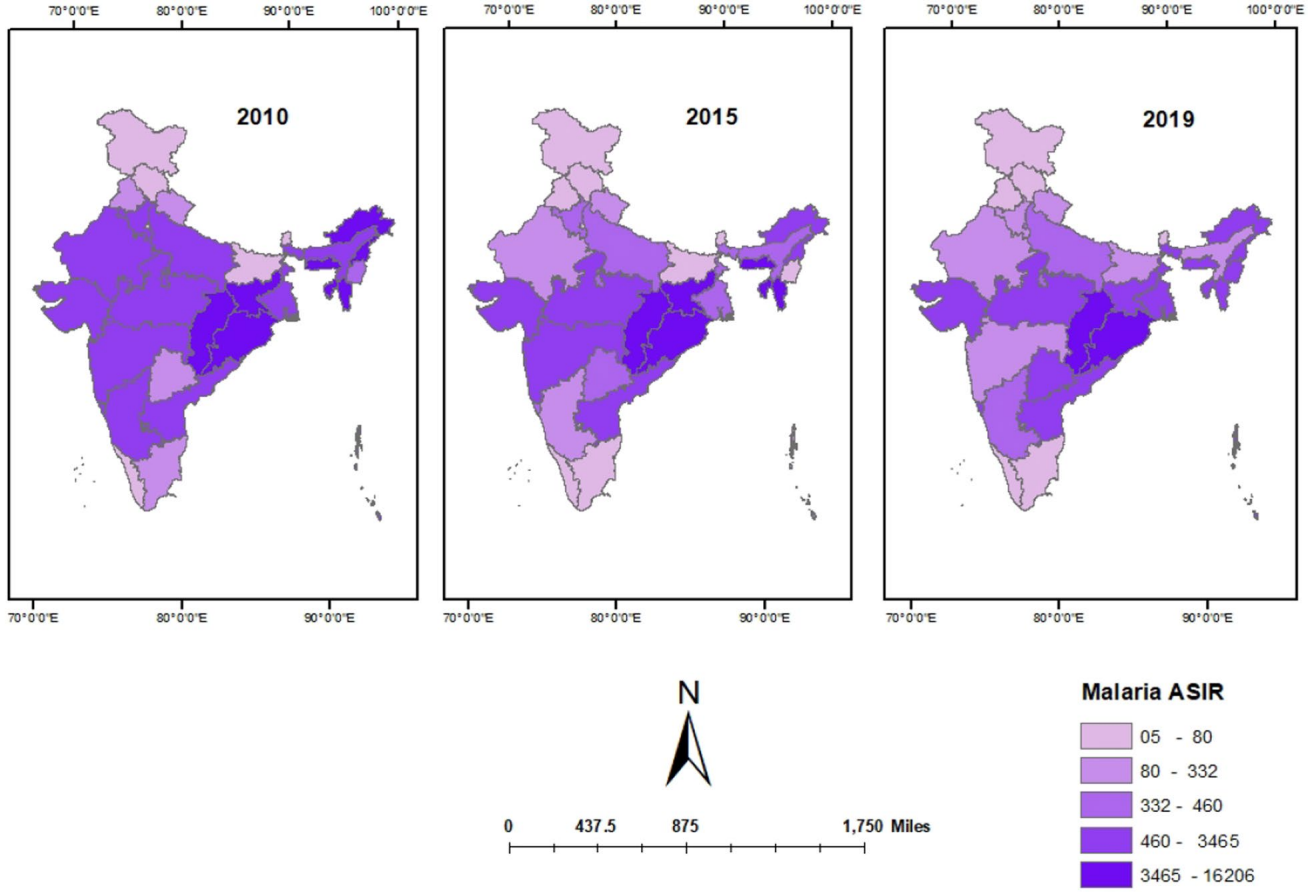
State-wisemap of malaria ASIR in different states of India in 2010, 2015, and 2019

Arunachal Pradesh had the highest ASIR of malaria in 2010 11,948.86 per 100,000 population followed by Mizoram 11,814.42 per 100,000 and lowest in Himanchal Pradesh 23.83 per 100,000 population followed by Delhi 25.17 per 100,000 population. Mizoram had the highest ASIR of malaria in 2015 16,206.09 per 100,000 population followed by Meghalaya 9944.92 per 100,000 and lowest in Delhi 4.69 per 100,000 population followed by Himanchal Pradesh 25.17 per 100,000 population. In 2019, Chhattisgarh had the highest ASIR of malaria in 4789.26 per 100,000 population followed by Odisha 4116.95 per 100,000 and lowest in Himanchal Pradesh 5.56 per 100,000 population followed by Sikkim 6.39 per 100,000 population (Fig. 2).

### Annual average precipitation and temperature variation by states in India (2010, 2015, 2019)

**Fig. 3:**
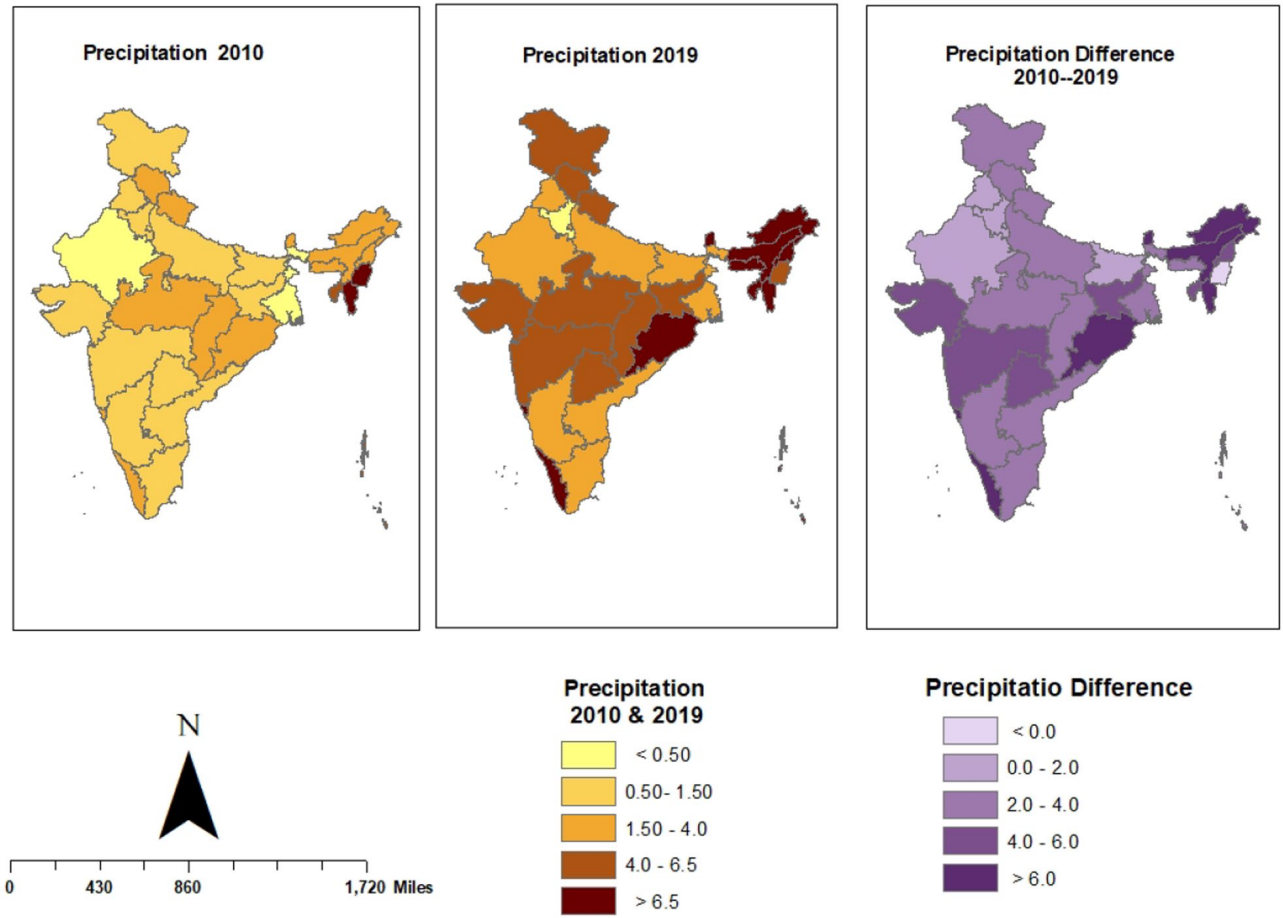
State-wise Annual Average Precipitation in India from 2010 to 2019 and Changes Over the Decade

In 2010, Maharashtra had the highest monthly precipitation at 15.46 mm, followed by Meghalaya with 7.22 mm and lowest in West Bengal with monthly precipitation 0.39 mm followed by Rajasthan 0.45 mm. By 2019, Meghalaya had the highest monthly precipitation at 18.17 mm followed by Arunachal Pradesh 12.54 mm and lowest in Haryana with monthly precipitation at 1.31 mm followed by Delhi 1.79 mm. The maximum increase in precipitation was observed in Meghalaya, from 7.22 mm to 18.17 mm per month, followed by Arunachal Pradesh, which saw an increase from 2.45 mm to 12.54 mm. While, the most notable decreases were in Maharashtra, where precipitation dropped from 15.46 mm to 4.54 mm per month, and in Haryana, which slight decrease from 1.17 mm to 1.31 mm per month (Fig. 3).

**Fig. 4:**
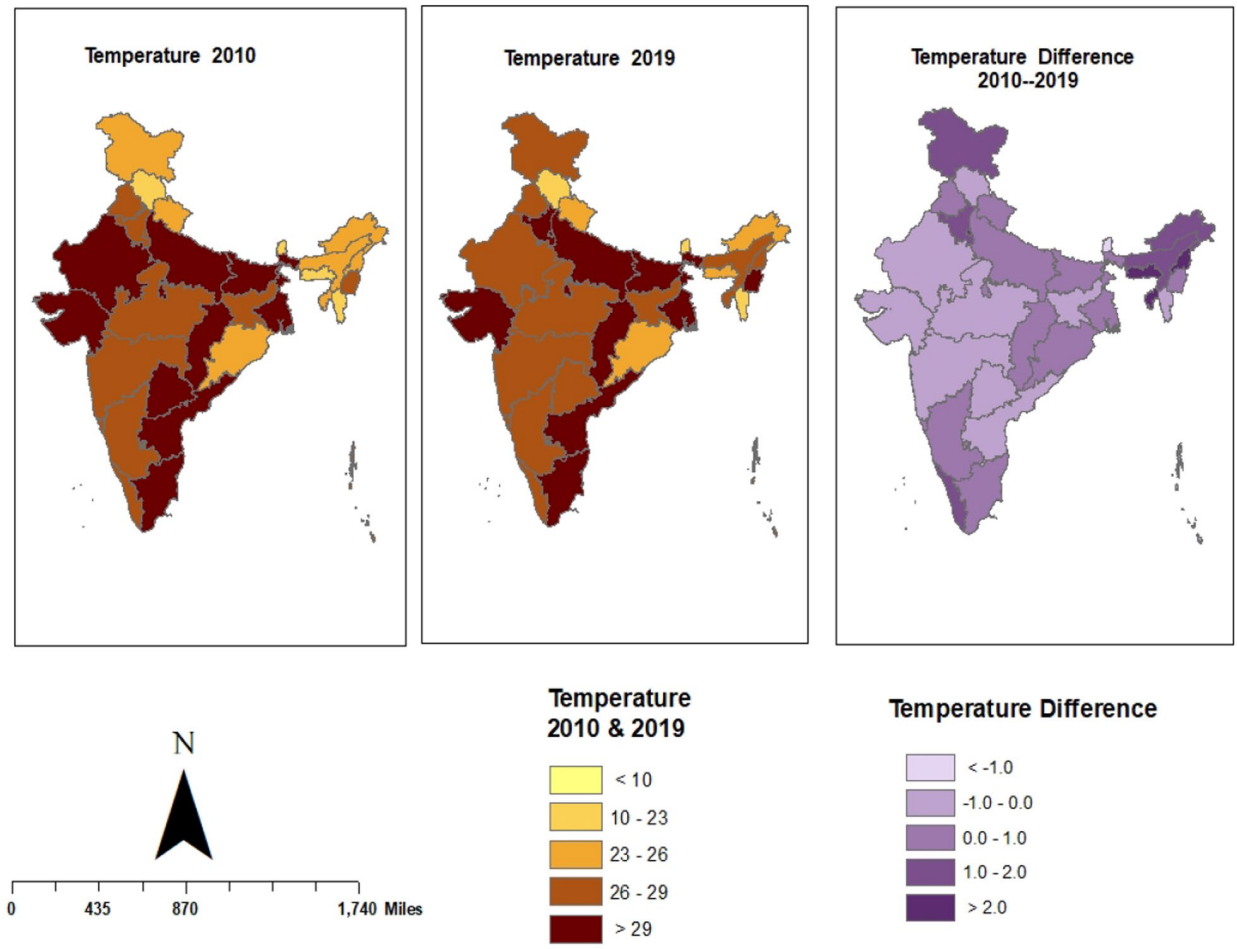
State wise Annual Average Temperature in India from 2010 to 2019, and their change between 2010 and 2019

In 2010, India's average annual temperature was noted at 24.79 °C. The highest temperatures were recorded in Andhra Pradesh, reaching 30.64 °C, with Tamil Nadu and Gujarat following closely at 30.31 °C and 29.85 °C, respectively. On the other hand, the lowest temperatures were found in Ladakh at − 8.87 °C, Sikkim at 14.51 °C, and Gujarat at 19.23 °C. By 2019, India's average temperature had risen to 26.33 °C. Delhi recorded the highest temperatures at 30.96 °C, with Tamil Nadu and Andhra Pradesh following closely at 30.87 °C and 30.52 °C, respectively (Fig. 4).

The pair-wise correlations among the fixed effects and the corresponding VIFs are sufficiently low to conclude that multicollinearity is not a concern for this model. The AIC and BIC values served as relative indicators of model performance, with lower values suggesting a better balance of complexity and fit. The proximity of AIC and BIC values in this analysis implies that the model, despite its complexity due to the inclusion of multiple fixed and random effects, does not overfit the data. The marginal R-squared value of 0.367 indicates that approximately 36.7% of the variance in ‘ASIR integer’ is explained by the fixed effects (‘Temperature Deviation’, ‘Normalized Precipitation’, and ‘HDI’) alone. The conditional R-squared, which is nearly 1, reveals that almost all the variability in `ASIR Integer` is accounted for when incorporating both fixed effects and random effects (`States` and `Year`). This exceptionally high conditional R-squared value is characteristic of models with substantial random effects components, demonstrating that the model is highly effective in capturing the complex data structure by integrating both observed predictors and unobserved heterogeneity (random effects).

### Marginal R^2^ (R^2^m) for fixed effects: 0.367 (variance explained by fixed effects only)

The model coefficients (Table 1) clearly illustrate the direct and multiplicative impacts of temperature deviations, precipitation changes, and HDI on malaria incidence rates. The results highlight the complex interplay of climatic factors and human development in managing and predicting Age-Specific Incidence Rate of malaria in different states of India.

**Table 1:** Fixed effects results with 95% confidence intervals

Variable	Coefficient	95% CI	Exp (Coeff.)	Std. Error	z-value	p-value	% Change
Intercept	2.39258	(0.850, 3.935)	–	0.78533	3.047	0.002314	–
Temperature deviation	0.49932	(0.488, 0.511)	1.647	0.00561	89	< 2e−16	+ 64.7% per °C increase
Normalized precipitation	0.01838	(0.008, 0.029)	1.0185	0.00516	3.562	0.000369	+ 1.85% per SD increase
HDI	3.33401	(2.911, 3.757)	28.02	0.2151	15.5	< 2e−16	+ 2702% per unit increase

The Table 1 shows each one-degree Celsius deviation from the baseline temperature of 28 °C, the ASIR of malaria is multiplied by approximately 1.647. This indicates a 64.7% increase in Age Specific Incidence of malaria per degree Celsius increase in temperature deviation, highlighting a substantial sensitivity to temperature changes. The Table 1 also indicates for each one standard deviation increase in normalized precipitation, the ASIR of malaria is multiplied by approximately 1.0185. This reflects an 1.85% increase in Age Specific Incidence of malaria per standard deviation increase in precipitation. Although the percentage increase is smaller compared to temperature, it still signifies a statistically significant positive relationship between increased precipitation and higher malaria transmission. The Table 1 illustrates for each unit increase in the HDI, the ASIR of malaria is multiplied by approximately 28.02. This dramatic increase suggests a strong positive association between HDI and reported malaria incidence, possibly reflecting better detection and reporting capabilities in regions with higher development indices.

The state-specific random effect values (SD: 4.2504) indicate substantial variability in malaria incidence across different states that are not captured by the fixed effects (Table 2). The high standard deviation relative to the mean incidence suggests significant disparities in malaria prevalence among states, possibly due to ecological, demographic, or health infrastructure differences which could not be captured by the Temperature, Precipitation and HDI only. The year-specific random effect (SD: 0.57) reflects annual variability in malaria incidence which affects all states but may vary year to year, potentially influenced by overarching climate trends, national malaria control policies, or other nationwide health interventions. Hence, it could be found that each factor contributes distinctly to shaping malaria dynamics, underscoring the need for integrated and adaptable public health strategies that consider environmental and socio-economic developments.

**Table 2:** Random effects results with 95% confidence intervals

Effect	Variance	Std. Dev	Random effects
States	18.0661	4.2504	Most positive: Jharkhand (+ 3.96), Most negative: Ladakh (− 20.60)
Year	0.3254	0.5704	Most positive: 2010 (+ 0.643), Most negative: 2019 (− 0.533)

AIC: 27,715.8; BIC: 27,732.0

Conditional R^2^ (R^2^c): 0.99 (Variance explained by full model)

**Fig. 5:**
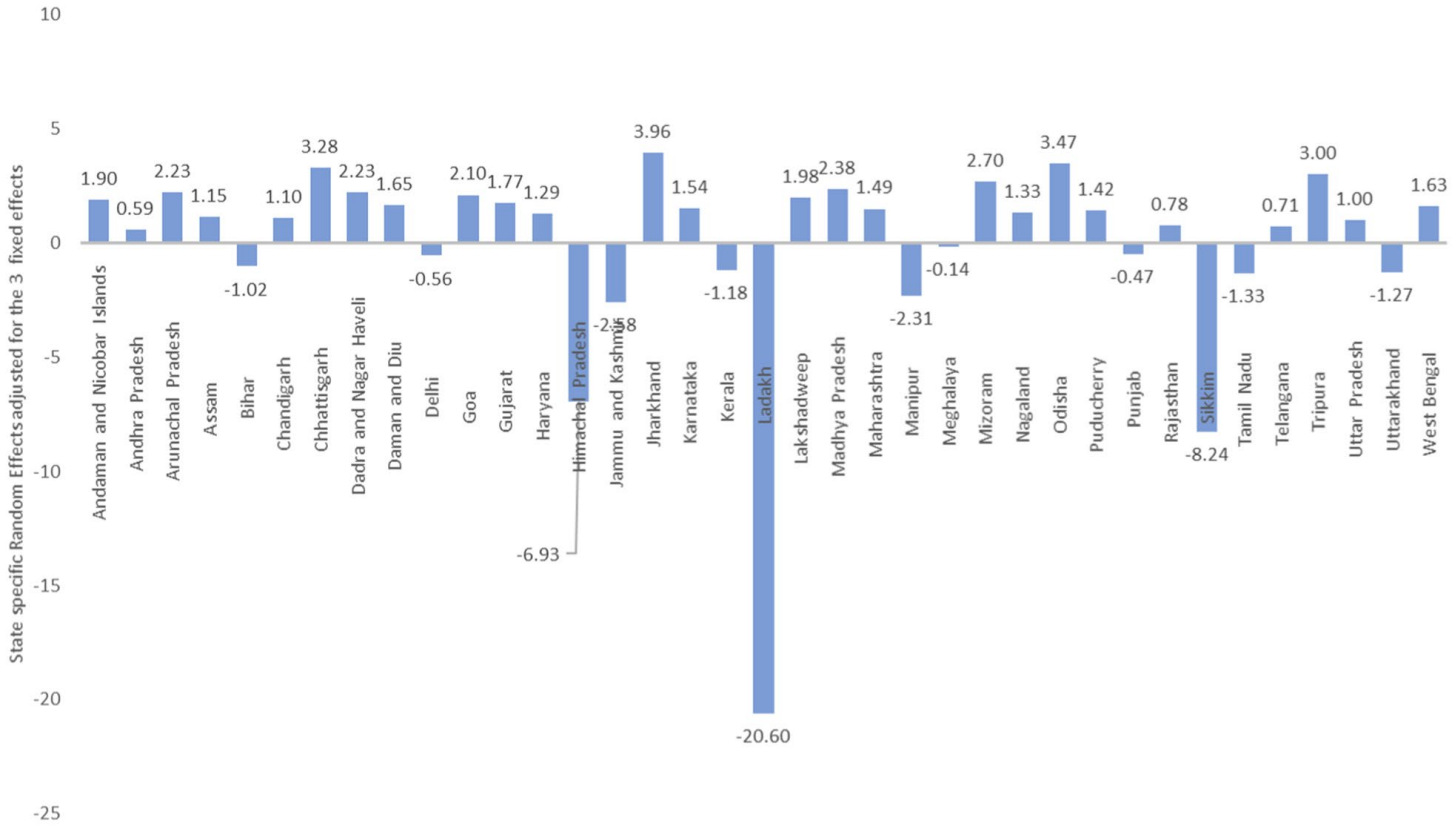
State-specific random effects adjusted for 3 fixed effects

Further analysis of the state-specific random effects (Fig. 5) indicates how much higher or lower the log of the Age-Specific Incidence Rate (ASIR) of malaria is in each state compared to the overall average (intercept), after adjusting for the effects of temperature deviation, normalized precipitation, and Human Development Index (HDI). A positive random effect for a state suggests that the ASIR of malaria is higher than the average predicted by the fixed effects alone. For instance, Chhattisgarh has a random effect of 3.28, indicating a significantly higher ASIR compared to the overall model intercept. This could be due to factors not captured by the fixed effects such as local ecological or socioeconomic conditions, or state-specific public health deficiencies or challenges. A negative random effect implies a lower ASIR of malaria than the average. Himachal Pradesh and Sikkim, with random effects of -6.93 and -8.24, respectively, have much lower ASIRs compared to what would be predicted by the fixed effects alone. This could reflect more effective malaria control measures, less suitable ecological conditions for mosquito breeding, or underreporting of cases. The extremely negative value for Ladakh (− 20.59) suggests a substantially lower incidence rate, likely due to its geographic and climatic conditions (other than temperature and precipitation) which are not conducive to mosquito breeding.

Similarly, the analysis of year-specific random effects shows the deviation in log ASIR of malaria from the overall average in specific years. For the year 2010 (0.64), the positive effect indicates that the incidence rate was higher than average in 2010, possibly due to less effective malaria control measures in place or a particularly warm and wet year conducive to mosquito breeding. For the other two years, 2015 (− 0.11) and 2019 (− 0.53), the negative effects suggest lower than average incidence rates, which could be attributed to improved malaria interventions, changes in environmental factors (other than the factors of Temperature and Precipitation), or other public health improvements over time which could not be captured by HDI alone.

The above results suggest that higher deviations from the baseline temperature and increased precipitation are associated with higher malaria incidence rates, controlled for HDI and spatial–temporal variability. The HDI's strong positive effect implies that regions with better human development metrics experience higher malaria incidence, potentially reflecting greater reporting accuracy or other socio-economic factors influencing disease prevalence. The analysis highlights the complex interplay of environmental and socio-economic factors in the epidemiology of malaria across different geographical and temporal scales in India.

## Discussion

The climatic heterogeneity across India, especially notable between the northern plains and southern peninsular regions, affects malaria transmission cycles and intervention success. Adaptive policies that incorporate climate change projections and their impact on vector ecology could be crucial in future malaria control and elimination efforts. For instance, increasing temperatures might expand the geographical range of certain vectors, necessitating expanded surveillance and control areas.

The intricate interplay of ecological, climatic, and socioeconomic factors delineates the complex landscape of malaria transmission across India, particularly in states like Odisha, Jharkhand, Madhya Pradesh, Chhattisgarh, and Maharashtra [40, 41]. The findings from the model, supported by empirical evidence from recent studies, highlight the pivotal roles of temperature, precipitation, and human development in influencing malaria dynamics. These elements, combined with state-specific characteristics such as topography and local vector species, necessitate tailored public health interventions that are responsive to these diverse conditions [40].

The states of Odisha, Jharkhand, Madhya Pradesh, Chhattisgarh, and Maharashtra collectively bear a significant burden of India's malaria cases. These regions are characterized by diverse ecological conditions that inherently support the breeding of malaria vectors. Temperature deviations from the optimal mosquito breeding range (around 28 °C) and increased precipitation are significant drivers of malaria transmission. Elevated temperatures are known to expedite the mosquito maturation process and the malaria parasite's development period, thereby increasing transmission rates, particularly noted in Chhattisgarh and Jharkhand. Similarly, heavy monsoon rains in Maharashtra and Odisha enhance mosquito breeding opportunities by creating extensive standing water, thus complicating vector control efforts [41].

Furthermore, the influence of altitude on malaria prevalence is particularly pronounced in the Western Ghats regions affecting Maharashtra and Madhya Pradesh, where higher elevations are associated with reduced vector density and lower transmission rates [42]. These findings align with the model’s predictions that climatic factors such as temperature and precipitation, which protect and preserve dense forest covers, critically influence malaria dynamics across these ecologically diverse settings.

The relationship between socioeconomic factors, particularly the Human Development Index (HDI), and malaria incidence is complex and multifaceted. Higher HDI unexpectedly correlates with increased malaria incidence, likely reflecting better disease surveillance and reporting capabilities in more developed areas such as urban centers in Maharashtra and Madhya Pradesh. It was found that urbanization affects malaria transmission dynamics. An Ahmedabad based study highlighted that in densely populated urban areas like Ahmedabad, where poverty and population density intersect with climatic variables, there is a structurally higher risk of malaria. This study provided insights into how urban environments facilitate the breeding of malaria vectors such as *Anopheles stephensi* and emphasized the role of socioeconomic factors like population density and poverty in urban malaria risk [43]. Conversely, the tribal and remote areas of Jharkhand and Chhattisgarh, despite their lower HDI, report high malaria incidence due to limited access to healthcare and other essential services. The association between higher HDI and increased reported cases of malaria paradoxically points to both the success in and the need for improving surveillance and healthcare responsiveness. More developed regions, often characterized by better healthcare facilities and reporting systems, reveal higher incidences of malaria, potentially reflecting more accurate data capture rather than higher transmission rates per se. This underscores the importance of enhancing health system capabilities in lower HDI regions, where underreporting and inadequate health infrastructure obscure the true burden of malaria and impede effective response strategies. The intra-state disparities in HDI within these states underscore the inequality in health resource distribution, impacting the efficacy of malaria control measures. These disparities are stark in tribal and rural areas, which are frequently overlooked in public health planning and resource allocation.

The significant regional variability in malaria incidence can also be attributed to the movement of populations across regions with varying endemicities, particularly from high to low transmission areas. It specifically mentions migrant workers who move between states, carrying the malaria parasite across geographical boundaries [42]. This mobility complicates the control of malaria as it contributes to the spread of the disease from low SDI high endemic states like Jharkhand and Chhattisgarh to high SDI states like Maharashtra, Gujarat, etc. Hence multilevel demographic events like population density, urbanization, and migration, in combination with ecological conditions, and climatic factors, underscore the necessity for localized malaria control strategies. States with high forest cover and tribal populations, such as Jharkhand and Chhattisgarh, require strategies that address the specific challenges posed by these environments, including the implementation of community-based vector control initiatives and the enhancement of local healthcare infrastructures. Similarly, urban areas in Maharashtra and Madhya Pradesh, affected by different vectors and higher population densities, might benefit from urban-specific health interventions and sanitation improvements to control vector populations.

Given the significant variability in malaria incidence driven by ecological and socioeconomic factors, targeted policy interventions are essential:

Targeted vector control and environmental management: Adapting vector control strategies to local climatic conditions can significantly enhance intervention effectiveness. This approach should integrate biological control measures and habitat manipulation, especially in areas prone to flooding or dense vegetation [41].

Infrastructure improvement and resource allocation: There is a critical need to strengthen healthcare infrastructure in low HDI regions and ensure equitable resource distribution. Enhancing medical facilities, improving diagnostic capabilities, and ensuring the availability of malaria prophylaxis are pivotal strategies [42].

Community-based interventions and education: Community-driven health education programmes that enhance preventive measures and promote better treatment-seeking behaviors can empower populations, particularly in rural and tribal areas [42].

Comprehensive surveillance systems: The development of robust surveillance systems that utilize both traditional reporting methods and modern technologies like geographic information systems (GIS) will facilitate timely and effective responses to malaria outbreaks and assist in strategic planning [42].

Intersectoral and interdisciplinary approaches: Encouraging collaboration across sectors such as water management, urban planning, and education, and integrating research findings into public health strategies can comprehensively address the socio-environmental determinants of malaria [43].

## Conclusion

The complex interplay of ecological, climatic, and socioeconomic factors shapes the malaria landscape in India. Supported by empirical research, empirical research supports the model, highlighting the need for region-specific malaria control strategies that are responsive to local variations. India's ambitious goal to eliminate malaria by 2027, which aligns with global targets but requires intensified efforts, particularly in high-burden states. Enhanced surveillance, community engagement, and state-specific interventions are crucial for effectively reducing malaria incidence. Moreover, national policies must strive to bridge healthcare disparities to achieve equitable health outcomes across all states. By addressing both immediate and systemic factors contributing to malaria transmission, India can progress significantly toward its goal of malaria elimination, ensuring a healthier, more equitable future for all residents.

## Data Availability

No datasets were generated or analysed during the current study.
